# A fully automated machine-learning-based workflow for radiation treatment planning in prostate cancer

**DOI:** 10.1016/j.ctro.2025.100933

**Published:** 2025-02-11

**Authors:** Jan-Hendrik Bolten, David Neugebauer, Christoph Grott, Fabian Weykamp, Jonas Ristau, Stephan Mende, Elisabetta Sandrini, Eva Meixner, Victoria Navarro Aznar, Eric Tonndorf-Martini, Kai Schubert, Christiane Steidel, Lars Wessel, Jürgen Debus, Jakob Liermann

**Affiliations:** a Klinik für Radioonkologie und Strahlentherapie Universitätsklinikum Heidelberg Germany; b Klinische Kooperationseinheit Strahlentherapie Deutsches Krebsforschungszentrum (DKFZ) Heidelberg Germany; c Nationales Zentrum für Tumorerkrankungen (NCT) Heidelberg Germany; d Heidelberg Institute of Radiation Oncology (HIRO) Universitätsklinikum Heidelberg Germany; e Klinik für Strahlentherapie und Radiologische Onkologie Kliniken Maria Hilf Mönchengladbach Germany; f Universitätsklinikum Lozano Blesa de Zaragoza Saragossa Spain

**Keywords:** Artificial intelligence, Fully automated radiation treatment planning, Prostate cancer, Auto segmentation in radiation oncology, Investigator-dependant variability, One-click radiation treatment planning

## Abstract

**Introduction:**

The integration of artificial intelligence into radiotherapy planning for prostate cancer has demonstrated promise in enhancing efficiency and consistency. In this study, we assess the clinical feasibility of a fully automated machine learning (ML)-based “one-click” workflow that combines ML-based segmentation and treatment planning. The proposed workflow was designed to create a clinically acceptable radiotherapy plan within the inter-observer variation of conventional plans.

**Methods:**

We evaluated the fully-automated workflow on five low-risk prostate cancer patients treated with external beam radiotherapy and compared the results with conventional optimized and inverse planned radiotherapy plans based on the contours of six different experienced radiation oncologists. Both qualitative and quantitative metrics were analyzed. Additionally, we evaluated the dose distribution of the ML-based and conventional radiation treatment plans on the different segmentations (manual vs. manual and manual vs. automation).

**Results:**

The automatic deep-learning segmentation of the target volume revealed a close agreement between the deep-learning based and expert contours referring to Dice Similarity- and Hausdorff index. However, the deep-learning based CTVs had a significantly smaller volume than the expert CTVs (47.1 cm3 vs. 62.6 cm^3^). The fully automated ML-based plans provide clinically acceptable dose coverage within the range of inter-observer variability observed in the manual plans. Due to the smaller segmentation of the CTV the dose coverage of the CTV and PTV (expert contours) were significantly lower than that of the manual plans.

**Conclusion:**

Our study indicates that the tested fully automated ML-based workflow is clinically feasible and leads to comparable results to conventional radiation treatment plans. This represents a promising step towards efficient and standardized prostate cancer treatment. Nevertheless, in the evaluated cohort, auto segmentation was associated with smaller target volumes compared to manual contours, highlighting the necessity of improving segmentation models and prospective testing of automation in radiation therapy.

## Highlights

• Clinically feasible RT plans by one-click ML-based workflow.
• ML-based RT plans within investigator-dependant variability.
• High potential to increase efficency and accuracy.

## Introduction

Implication of artificial intelligence is a very promising tool to increase efficiency and consistency in radiation treatment (RT) of prostate cancer as one of the most common malignancies affecting men worldwide [1], [2], [3], [4], [5]. RT planning can roughly be divided into two main parts. First, the segmentation of target volumes (TVs) and organs at risk (OARs). Second, after contouring is completed, the RT plan is performed by a Radiation Therapy Technologist (RTT). Guidelines have been established to standardize the contouring of TVs. However, TV delineation in RT of prostate cancer remains one of the weaknesses of radiotherapy, with significant inter-observer variability persisting [6], [7], [8], [9], [10]. Consequently, developing and integrating machine-learning (ML) protocols that produce a consistent and anatomically accurate organ segmentation is highly warranted.

Recent studies demonstrate the potential of ML-based approaches in improving the accuracy and efficiency of the RT planning process. Deep learning algorithms that automatically segment TVs and OARs lead to more consistent and reliable contours compared to manual methods​​. Furthermore, the clinical integration of these technologies could reduce the time and effort required for the RT planning process, allowing for more personalized and adaptive treatment strategies [11], [12], [13], [14], [15]. This might lead to a more accurate dose application and less toxicities.

In a prospective study, McIntosh et al. deployed and evaluated a random forest algorithm for curative-intent RT planning for prostate cancer that could be implemented in the clinical workflow. Therefore, they demonstrated the feasibility of a ML-generated RT plan based on human-manually defined segmentation and achieved significant time efficiencies compared to the standard manual work-flow [16], [17].

In the current study, we analyze the clinical feasibility of a fully-automated ML-based one-click workflow combining ML-based segmentation and ML-based RT planning [18], [19]. To initialize the work-flow, solely a native CT scan and the pre-defined treatment concept are needed. We aim to achieve a fully automatically generated RT plan within the interobserver variation and that is clinically acceptable.

## Methods

### Automated machine-learning radiation treatment planning workflow

**Fig. 1:**
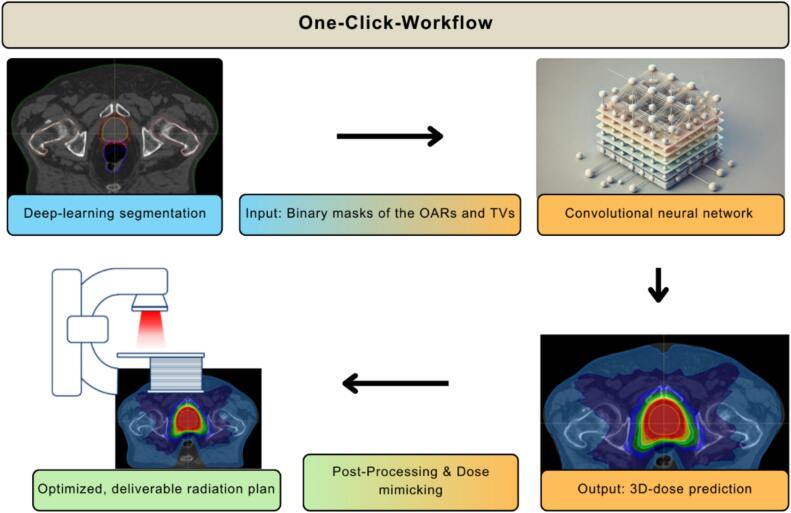
A simplified illustration of the one-click-workflow for radiation treatment planning. The workflow consists of multiple steps in the Treatment Planning System using deep-learning segmentation, machine-learning based planning model and post-processing steps, connected by the scripting tool.

The fully automated workflow for RT planning was developed within the treatment planning system (TPS) RayStation (v11B, RaySearch Laboratories), using the built-in deep-learning based segmentation tool (RLS Male Pelvic v2.0.0) and an in-house modified version of the machine learning planning model RSL-Prostate-6000. This model consists of a fixed training data set with clinically approved treatment plans and utilizes a convolutional neural network to predict a dose prediction for a new patient, followed by a dose mimicking algorithm to generate deliverable treatment plans. To account for clinic specific characteristics, a post-processing of the predicted dose and the dose mimicking process can be configured by the user. The two main components are linked and complemented with tasks like algebra operations to create the TVs using the scripting interface within the TPS. After importing the planning CT scan to the TPS, the workflow can be initialized with a single mouse click and creates a treatment plan in approximately 10 min without any human intervention. A simplified illustration of the workflow is given in Fig. 1 and an extended chart is added in the supplementary material (2.1).

### Imaging protocol

The quality of autosegmentation depends on CT image quality. In this study, CT images were acquired using the in-house standard pelvis-imaging protocol, that is used for RT of the prostate. CT images (512x512 pixels) were acquired (120 kV, 300mAs) with a Somatom Sensation Open or Somatom Sensation Confidence (Siemens Healthineers, Erlangen, Germany). All images were acquired in the head-first supine position. The images used for ML-based segmentation were reconstructed with a 3-mm slice thickness, 3-mm slice interval, a 50 cm reconstruction diameter and the filtered back projection B40s.

### Treatment protocol

We created a workflow for a treatment schedule with 76.5 Gy in 34 fractions. The total dose is prescribed to the median with the goal to achieve at least 95 % dose coverage of at least 95 % of the PTV. A maximum dose of up to 107 % was deemed acceptable. The used dose constraints are shown in the supplementary material (2.2).

Five low-risk prostate cancer patients were irradiated from 2020 to 2022 with 76.5 Gy in 34 fractions. The CTV of these patients was restricted to the prostate (seminal vesicles not included) due to low-risk prostate cancer classification. In two cases, a pelvic MRI was available and could be used for manual contouring. The ML-based contouring was based on the native pelvic CT only.

### Contour analysis

Segmentation of the TV and OARs was individually performed by six experienced radiation oncologists (experience defined as having planned at least 100 prostate cancer RT-patients) and the ML-based autosegmentation. This resulted in a total of six conventional manual expert contour (EC) datasets (including the original contour dataset) and one deep-learning-based contour (DC) dataset per patient for evaluation. To analyze the DCs and the interobserver variability of the TV, Dice-Similarity-Coefficient and the max. Hausdorff-Index were used [20], [21], [22], [23].

DSC = 2(TV_a_ ∩ TV_b_ / TV_a_ + TV_b_)

HD = max {dHD(a,b), dHD(b,a)}

We compared the mean of the values from all manual PTVs with the mean of the ML-based PTVs. A subgroup analysis for every patient was calculated.

For statistical analysis, GraphPad Prism v.10.2.2 was used to perform repeated-measures one-way ANOVA and two-sided *t*-test analysis on the results. The difference was defined statistically significant with p < 0.05.

### Comparison of treatment plan quality

**Fig. 2:**
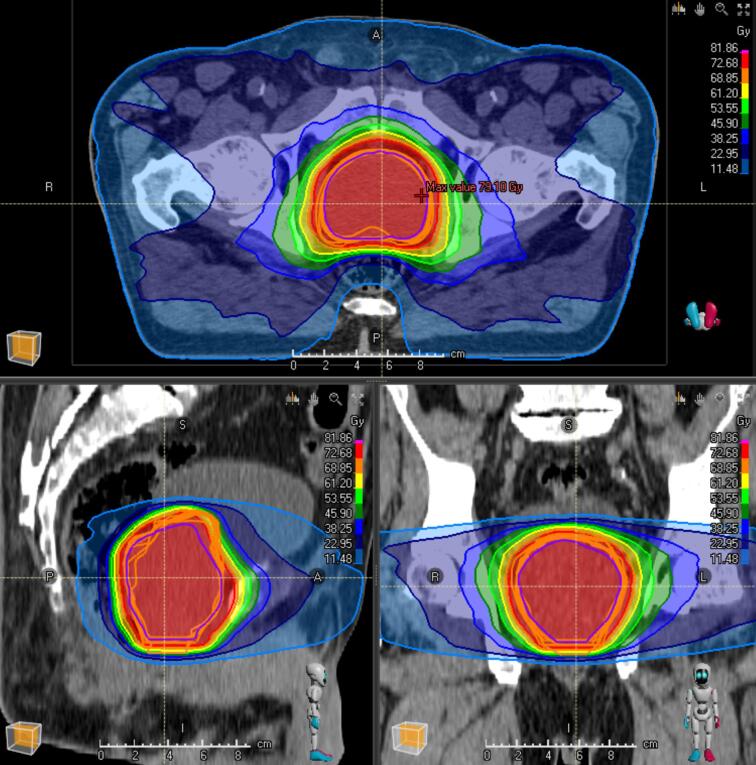
Dose distribution of ML-based RT plan. The image shows the dose distribution of the ML-based RT plan of the different CTVs. The ML-based CTV (purple) and the manual contoured CTVs (orange) are demonstrated.

For each conventional contour dataset, a manual volumetric modulated arc therapy (VMAT) plan using two full-arcs with 6 MV was generated. VMAT plans for the DCs were created automatically according to the above-mentioned workflow. All treatment plans were reviewed and approved by a board-certified radiation oncologist to ensure their clinical accuracy and safety. To evaluate and compare the conventional and ML-based treatment plans, we used qualitative and quantitative metrics. For a quantitative evaluation, we analyzed the dose distribution in each contour dataset using the respective other treatment plans. Through this, we could evaluate the dose distribution statistics of each plan in each contour dataset to compare the quantitative metrics. We assessed the V_95_, D_95_, D_50_, D_mean_, D_2_, conformity index (CI) and homogeneity index (HI) for CTV and PTV. The conformity index used is defined as $CI=\frac{Intersection\;of\;PTV\;and\;V95\%}{V95\%}$ (V_95%_=-volume with minimum of 95 % of the prescription dose). Homogeneity index is defined as $HI=\frac{D95}{D5}$. Additionally, we examined the dose exposure to the bladder, rectum, and femoral heads. This allowed us to make a statement about the dose coverage of the different contours by each plan. On the other hand, we evaluated the dose coverage of each contour set by the other treatment plans. Fig. 2 illustrates the dose distribution of a ML-based RT plan, also showing the different CTV contours (orange = manual CTVs; green = ML-based CTV).

The dose coverage of each contour was compared to reference values. The reference values were defined as the dose parameters of the contours, which were used for the RT planning of the evaluated RT plan. This provides an assessment of each individual plan as well as each contour dataset.

A calculation of the mean and standard deviation of the dose indices and of the deviation from the reference values was performed. Moreover, a 95 % confidence tolerance interval (tolerance interval = μ ± k × μ × CV (μ = mean; CV = coefficient of variation, k = 1.96)), was calculated.

To evaluate the interobserver variability, we analyzed the conventional plans among themselves. To make a statement about whether the deviations of the RT plans from the ML-based workflow are within the interobserver variability, we used the aforementioned tolerance interval.

GraphPad Prism v.10.2.2 was used to perform a descriptive statistical analysis and Mann-Whitney test. The difference was defined statistically significant with p < 0.05.

The study has been approved by the Ethics Committee of Heidelberg University (S-193/2024).

## Results

### Segmentation analysis

**Fig. 3:**
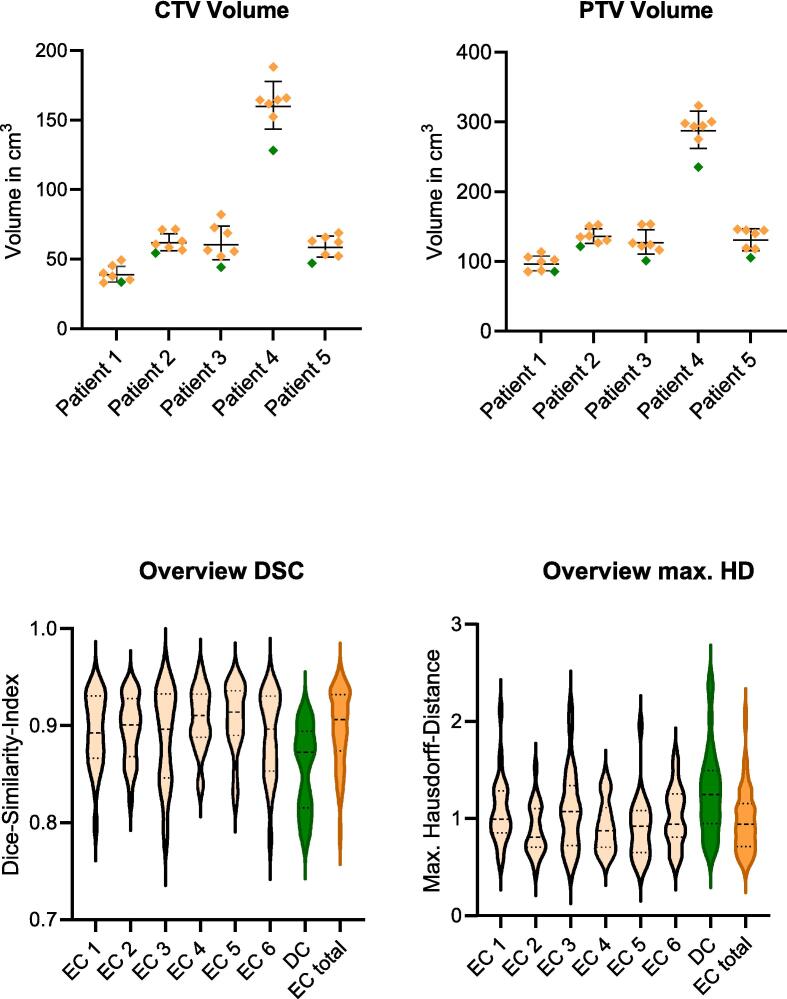
Comparison of CTV and PTV volumes of ECs and DCs. The upper graphs illustrate differences in the volume for CTV and PTV with a 95%-confidence interval. The ECs were depicted in orange, the DCs in green. The lower graphs provide an overview of DSC and max. HD for each contour compared to the entirety of the ECs.

The volumes of the ML-based CTVs were significantly smaller than the manual contoured CTV with a median of 47.1 cm^3^ vs. 62.7 cm^3^ (p < 0.05). Due to the same approach to create the PTV margins the ML-based PTVs were also significantly smaller than the manual contoured PTVs with 105.7 cm^3^ vs. 138.4 cm^3^ (p < 0.05). Fig. 3 provides a graphic overview of the volume differences. In a qualitative analysis both, the ECs and DCs seemed anatomically correct within common interobserver variabilities. In a slice-by-slice analysis of the CTV, differences in contouring were particularly evident at the transition to the penile bulb and the seminal vesicles. The bladder volume was 260.6 ± 61.6 cm^3^ vs. 263.1 ± 66.2 cm^3^ (EC vs. DC). The rectum volume was 78.7 ± 35.1 cm^3^ vs. 85.1 ± 34.6 cm^3^ (EC vs. DC). There was no statistically significant difference between the manual and ML-based volumes of the bladder and rectum. The DCs were reproduced consistently when recontoured by the ML-based contouring (data not shown).

### Dice-Similarity-Index (DSC)

The comparison of the means ± standard deviations of the DSC showed no significant differences between the ECs (p = 0.38). In contrast, the DCs demonstrated statistically significant differences compared to the ECs (EC 0.9 ± 0.04 vs. DC 0.86 ± 0.04; p < 0.001). A post-hoc analysis using Tukey’s multiple comparison test revealed that the deviations of the DC were significant for each EC.

### Max. Hausdorff-Distance

The comparison of the means ± standard deviation of the max. Hausdorff distance showed a significant difference between the ECs (p = 0.015). A post-hoc analysis using Tukey’s multiple comparison test revealed that the deviation was only significant between EC1 and EC2. The DCs demonstrated statistically significant differences compared to the EC (EC 0.98 ± 0.33 vs. DC 1.27 ± 0.45; p < 0.001). A post-hoc analysis using Tukey’s multiple comparison test revealed that the deviations were significant for three of six EC.

### Expert review

All RT plans met the in-house standard dose constraints and were approved by a board-certified radiation oncologist.

### Dosimetric analysis

In the initial assessment of the ML-based RT plans, it was shown that the aforementioned dose parameters for evaluating the dosimetric coverage of the TVs were within the 95 % confidence interval of the investigator-dependent variation of the manual plans (Table 1). This applies to the coverage of the different manually contoured TVs by the ML-based RT plans as well as the dose coverage of the DCs by different manual RT plans.

**Table 1:** Overview of deviations of dosimetric parameters. The table shows the means ± standard deviations and a tolerance interval of the dosimetric coverage of a structure by the other manual plans and the ML-plans compared to the plan associated to the structures.

	**95 %-Confidence-interval Manual**	**Manual M ± SD**	**ML M ± SD**	**p-value**
CTV ΔV_72.68Gy_ [%]	[-2.26; 1.59]	−0.33 ± 0.95	−1.65 ± 2.2	<0.001
PTV ΔV_72.68Gy_ [%]	[-18.25; 6.51]	−5.87 ± 5.64	−15.03 ± 6.82	<0.001
PTV ΔD_95_ [%]	[-26.34; 13.33]	−6.49 ± 10.25	−14.77 ± 11.38	<0.001
PTV ΔD_mean_ [%]	[-3.69; 1.09]	−0.99 ± 1.38	−3.19 ± 3.42	<0.001
PTV ΔHomogeneity-index	[-0.34; 0.04]	−0.08 ± 0.13	−0.19 ± 0.15	<0.001
PTV ΔConformity-index	[-0.2; 0.09]	−0.05 ± 0.08	0.03 ± 0.04	<0.001
Bladder V_50Gy_ [%]	[5.71; 28.49]	14.8 ± 6.99	9.42 ± 2.95	<0.001
Rectum V_50Gy_ [%]	[12.56; 25.97]	19.26 ± 3.42	17.8 ± 3.16	0.01

### Dose distribution in ECs by each RT plan

**Fig. 4:**
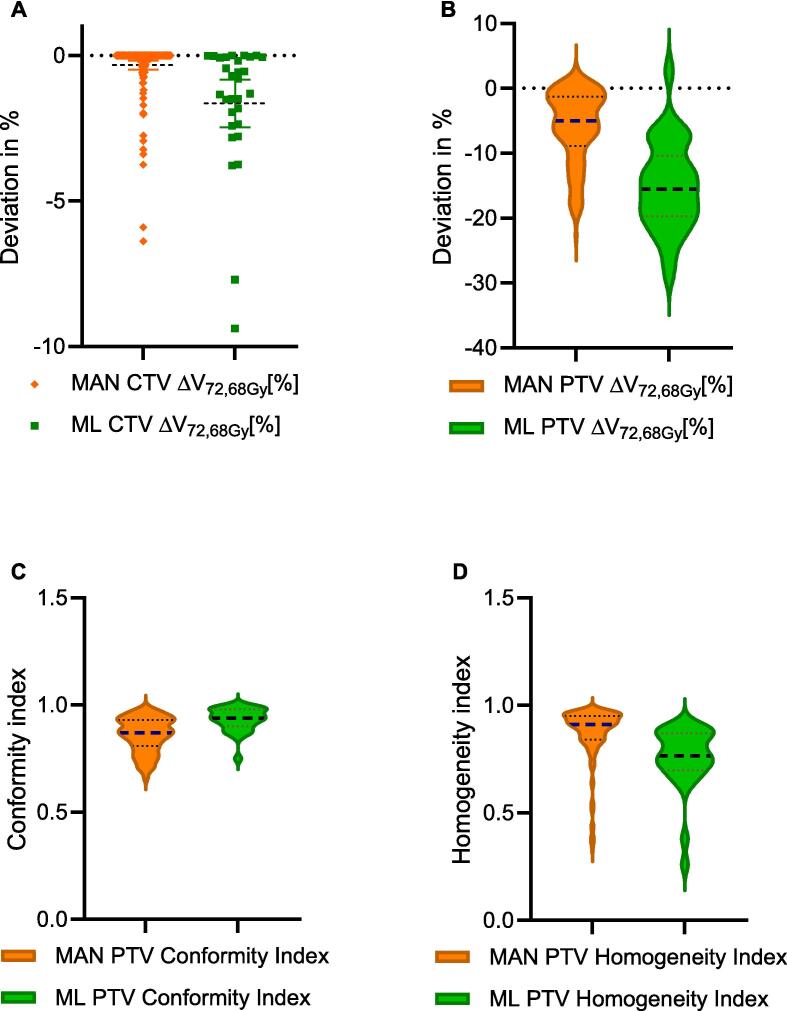
Comparison of target coverage between manual and ML-based RT-plans. To compare the CTV an PTV coverage, the deviations from the reference value are shown (A; B) and the absolute indices to compare CI and HI (C; D).

In most cases, evaluating the dose statistics of the different RT plans on the different contour datasets resulted in neglect of the dose constraints. This affected both the manual and the ML-based plans. The deviations (all patients) of the evaluated dosimetric parameters of the individual contours from the reference value (see above) were statistically significant in all analyzed parameters except for D_2_ in the CTV (Table 1). Smaller deviations to the reference value of the manual plans than ML-based plans were seen for V_72.68Gy_ [%] of the PTV with −5.9 ± 5.6 % vs. −15.03 ± 6.82 % (MAN vs. ML) and for the CTV with −0.33 ± 0.95 % vs. −1.65 ± 2.2 % (MAN vs. ML). Fig. 4 illustrates the differences of dose coverage in the TV. The difference of the D_95_ [%] in the PTV was also smaller in the manual plans with 6.49 ± 10.25 % vs. −14.77 ± 11.38 % (MAN vs. ML). CI in the PTV was closer to 1.0 in the ML-based RT plans. HI in the PTV was closer to 1.0 in the manual RT plans. The differences of the other dose parameters for the CTV were all in a range of < 5 %.

**Fig. 5:**
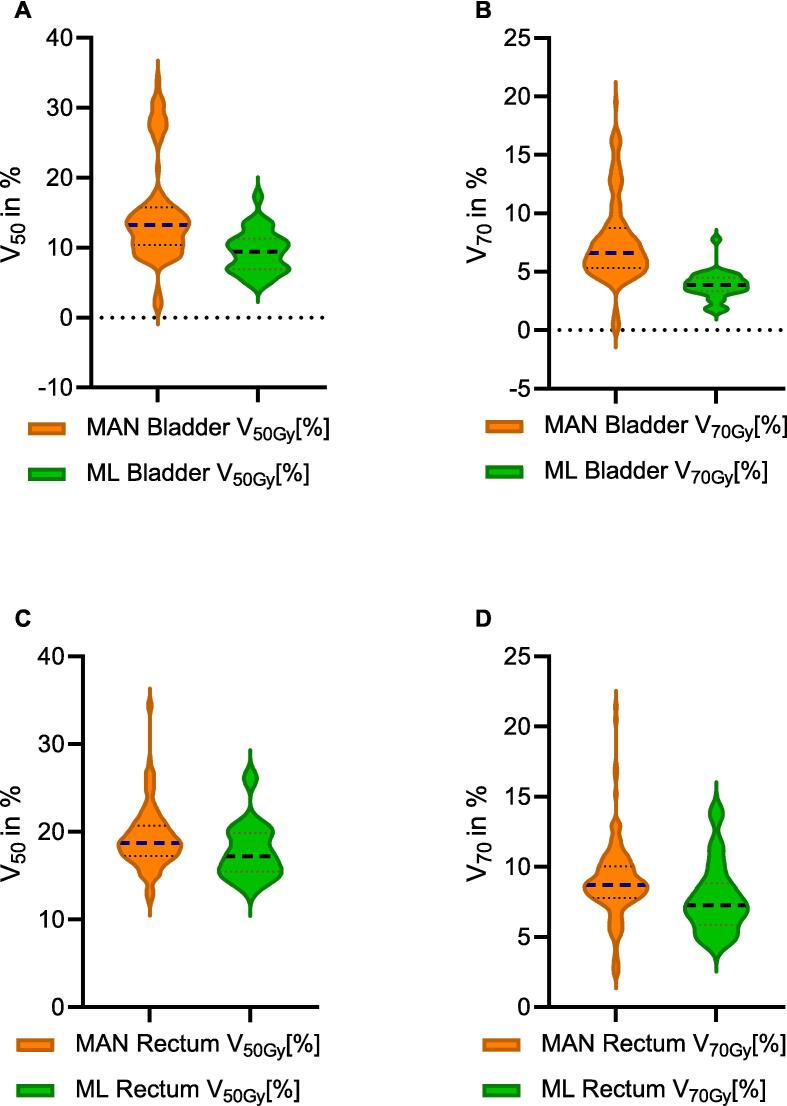
Comparison of dose exposure to bladder and rectum between manual and ML-based RT-plans. To compare the dose parameters V_50Gy_ and V_70Gy_ of the bladder (A; B) and V_50Gy_ and V_70Gy_ of the rectum (C; D).

The dose exposure of the OAR (bladder, rectum) was statistically significant lower in the ML-based RT plans. V_50Gy_ [%] of the bladder was 14.8 ± 6.99 % vs. 9.42 ± 2.95 % (MAN vs. ML) and V_70Gy_ [%] was 7.54 ± 3.46 % vs. 3.85 ± 1.16 % (p < 0.001; Fig. 3). V_50Gy_ [%] of the rectum was 19.24 ± 3.44 % vs. 17.8 ± 3.16 % (MAN vs. ML) and V_70Gy_ [%] was 8.94 ± 2.66 % vs. 7.75 ± 2.47 % (p = 0.02; Fig. 5).

Dose exposure to the femoral heads was similar for MAN and ML-based RT plans and met the dose constraints.

### Dose distribution in each contour by manual RT plans

There were no statistically significant differences for the evaluated dose parameters in the CTV and OAR between the ECs and DCs. The mean deviation from the reference value for V_72.68Gy_ [%] and D_95_ [%] in the PTV were statistically significant lower in the DCs with −5.87 ± 6.32 % vs. −0.93 ± 3.14 % and −6.51 ± 10.12 % vs. −0.77 ± 3.69 % (EC vs. DC; p < 0.001). All other differences were < 2 %.

## Discussion

The aim of this study was to prove that deviations by ML-based planning were within the interobserver variation. The results of our study illustrate that the fully ML-based RT plans exhibit a dose coverage of the manual TVs of the five patients within the range of investigator-dependent variation of conventionally manually created treatment plans. Therefore, the ML-based workflow for RT planning can provide clinical acceptable and to the conventional manual RT plans comparable RT plans. However, it should not be neglected that the ML-based RT plans demonstrated significantly lower dose coverage of the manual TVs, especially in the PTV, than the manual plans among themselves. Retrospectively, that is the logical consequence of overall smaller TVs in the ML-based workflow. This is partly due to the difficult delineation of the prostate especially in the area of the basis of the seminal vesicles and at the apex in CT imaging. Furthermore, the used autosegmentation of the TV was not trained with in-house data. As a consequence, systematic delineation differences could also be due to inter-institutional differences in TV definition. Ultimately, it cannot be clearly determined which of the contours is better − especially as no contour was obviously contoured anatomically wrong as approved by expert review. When comparing two clinical acceptable RT plans with anatomically correct organ delineation, it partly remains a decision of personal preference which one is better. Looking to the OAR, the ML-based RT plans spared the OAR significantly better than the conventional plans, whereby the ML-based delineation of the bladder and rectum were similar to manual ones. This is likely due to the smaller CTVs.

Compared to other studies examining deep-learning autosegmentation, the variation between the different contours (CTV) is rather low with an average DSC of 0.91 and a max. HD of 0.98 mm for EC to EC and a DSC of 0.86 and max. HD of 1.27 mm for EC to DC. The average difference between the interobserver variability and the DCs is within 0.05 for DSC and 3.0 mm for HD. In the existing literature the average DSC comparing ECs to ECs of the prostate is in a range of 0.8 to 0.9 [24], [25]. The average DSC comparing DCs to ECs were in a range of 0.79 to 0.84 in various other studies and the 95 %HD < 1.6 mm [25], [26], [27], [28]. Compared to these data, the used contouring model provides DCs with a small deviation to the EC in the range of EC to EC variability.

The fact that the evaluation of updated dose statistics of a RT plan on a different contour data set showed a neglect of the dose constraints in most cases, emphasizes the existing problems of inter-observer variation. Several studies have already demonstrated that an ML-based contouring of the relevant structures followed by manual optimization leads to a reduction in interobserver variation in prostate cancer [13], [14], [29], [30]. However, in this case the smaller CTV of the ML-based contouring leads to a reduced dose coverage, which might have the biggest impact on the quality of the radiation treatment plans. By training the deep-learning based segmentation model by our own with ECs followed the in-house standard based on different contouring guidelines, we would expect a stronger agreement of the ECs and DCs as well as the CTV and PTV dose coverage [10], [30], [31], [32], [33]. At the current time, the immutable and unknown training dataset of the deep-learning-based segmentation tool is a disadvantage of this auto-segmentation tool. On the other hand, it provides more consistent and uniform segmentations compared to manual contours. To reduce the interobserver variation even more a multi-institutional trained algorithm would be useful.

A limitation of this study is the small patient number of only five patients. For each of the 5 patients, we created six manual plans as well as one ML-based plan, resulting in a total of 35 different plans, all of which were compared with one another. But even in this small cohort, we observed occasional outliers among the manual plans regarding the dose coverage of the TV. Since the dose coverage was primarily reduced in the PTV and the CTV changed by less than 5 % in most cases, this raises questions regarding the clinical consequences of the interobserver variability.

Another aspect is that all selected patients had no anatomical peculiarities. Particularly unfavorable positioning of bowel loops leads to problems in the use of the automated workflow. It was also not tested to create a RT plan in atypical situations e.g. for patients with hip prostheses. Using this workflow with further optimization and validation in clinical practice is currently only possible with an expert review. Nevertheless, it could provide a time saving tool in treatment planning in patients with “regular” anatomy.

In 2021, McIntosh et al. already demonstrated that the ML-based RT planning algorithm used in the present analysis delivers clinically acceptable treatment plans in over 85 % of cases [16]. In the current study, we took it a step further by combining automatic ML-based RT plan optimization with automatic contouring of TVs and OARs. Given the well-known relatively high interobserver variability in prostate contouring, we initially considered contouring as the crucial factor for plan quality [34], [35], [36]. Therefore, we calculated the final ML-based treatment plan on multiple ECs and compared the dose distribution with the interobserver variability. Our results show that the plans fall within the range of interobserver variability considering the quantitative parameters [37], [38].

## Conclusion

In summary, we were able to demonstrate comparable deviations of the ML-based planning to the investigator-dependant variability. As we observed significant differences of the size of the TVs compared to the manual delineated ones, we see a necessity of improvement in autosegmentation. Nevertheless, this workflow is clinically feasible has the potential to drastically reduce the time required for RT planning.

## Declaration of Competing Interest

The authors declare the following financial interests/personal relationships which may be considered as potential competing interests: Juergen Debus received grants from Merck Serono GmbH, Accuray Incorporated, RaySearch Laboratories AB, Vision RT limited, Siemens Healthcare GmbH, Quintiles GmbH and PTW-Freiburg Dr. Pychlau GmbH outside the submitted work in the last 36 months. Jakob Liermann received travel fees from RaySearch Laboratories AB and Micropos Medical and speaker's fee from Accuray Incorporated.
