# Supplementary material for: A fully automated machine-learning-based workflow for radiation treatment planning in prostate cancer
Source: Clin Transl Radiat Oncol. 2025 Feb 11;52:100933. doi: 10.1016/j.ctro.2025.100933 (PMC11871478; doi:10.1016/j.ctro.2025.100933)
Supplement: Supplementary Data 1 [file mmc1.docx]

Supplementary material

2.1. One-Click-Workflow


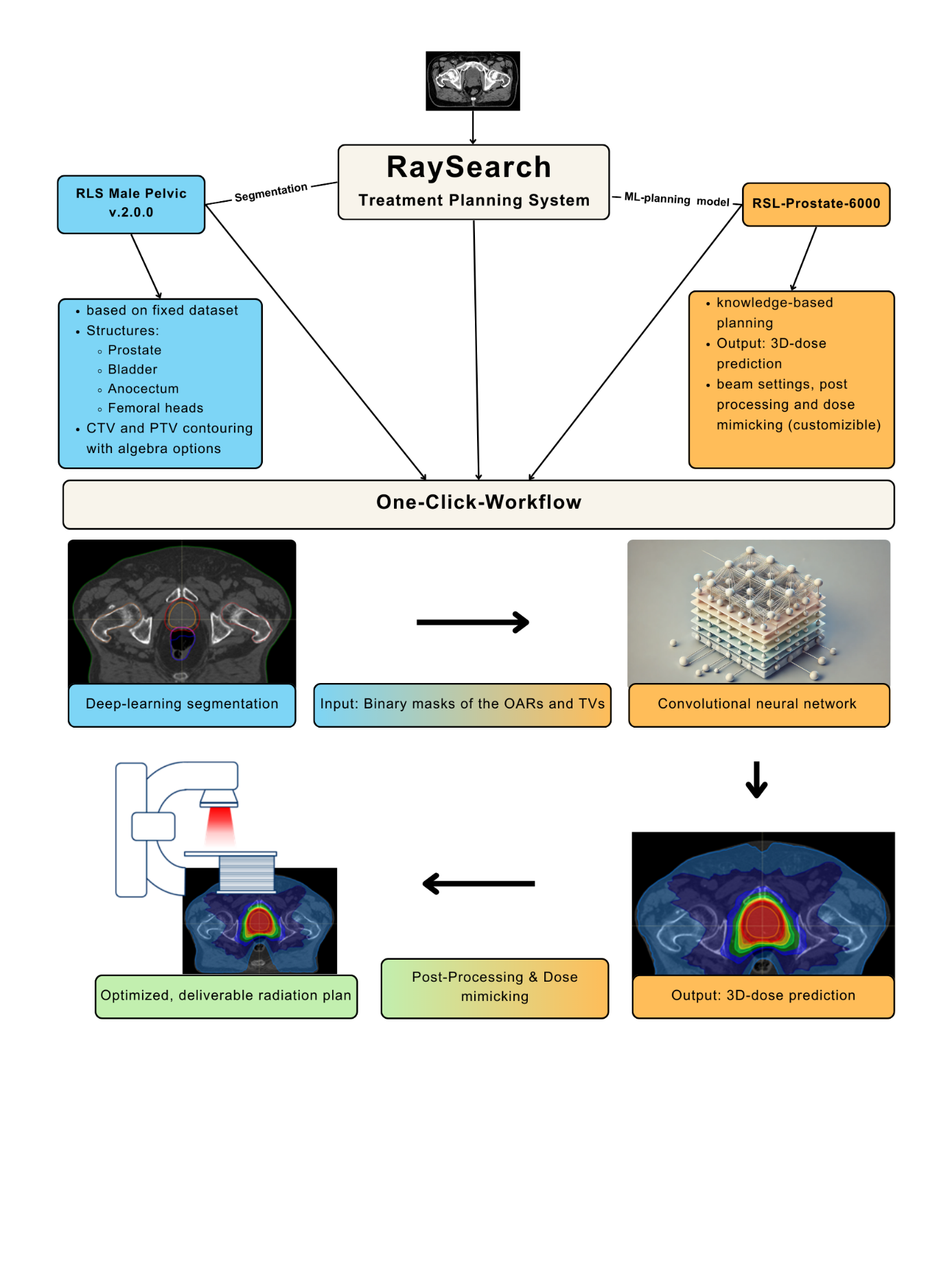


2.2 Dose Constraints

| Organ | Constraint |
| --- | --- |
| PTV Prostata | Min. 95.0 % at 72,68 Gy |
| Rectum | Max. 0.03 ccm at 78.0 Gy |
| Rectum | Max. 50.0 % at 50.0 Gy |
| Rectum | Max. 35.0 % at 60.0 Gy |
| Rectum | Max. 25.0 % at 65.0 Gy |
| Rectum | Max. 20.0 % at 70.0 Gy |
| Rectum | Max. 15.0% at 75.0 Gy |
| Bladder | Max. 35.0 Gy at average |
| Bladder | Max. 0.03 ccm at 71.0 Gy |
| Bladder | Max. 50.0% at 60.0 Gy |
| Bladder | Max. 35.0% at 70.0 Gy |
| Bladder | Max. 25.0% at 75.0 Gy |
| Bladder | Max. 15.0% at 77.0 Gy |
| Bowels | Max. 0.03 ccm at 52.0 Gy |
| Bowels | Max. 200 ccm at 45.0 Gy |
| Femoral head left | Max. 25.0 Gy average |
| Femoral head right | Max. 25.0 Gy average |
